# The Impact of COVID-19 Infection on Patients with Severe Chronic Pulmonary Hypertension: A Prospective Study from a Single Referral Center

**DOI:** 10.3390/medicina60050750

**Published:** 2024-04-30

**Authors:** Virginija Rudienė, Lina Kaplerienė, Monika Laukytė-Slėnienė, Dovilė Žebrauskienė, Vaida Averjanovaitė, Virginija Šileikienė, Ingrida Zeleckienė, Mindaugas Matačiūnas, Lina Gumbienė, Eglė Grigonienė

**Affiliations:** 1 Clinic of Cardiac and Vascular Diseases, Faculty of Medicine, Institute of Clinical Medicine, Vilnius University, 08661 Vilnius, Lithuania; lina.kapleriene@santa.lt (L.K.); monika.laukyte@santa.lt (M.L.-S.); lina.gumbiene@santa.lt (L.G.); egle.grigoniene@santa.lt (E.G.); 2 Clinic of Chest Diseases, Immunology and Allergology, Faculty of Medicine, Institute of Clinical Medicine, Vilnius University, 03101 Vilnius, Lithuaniavirginija.sileikiene@santa.lt (V.Š.); 3 Department of Radiology, Nuclear Medicine and Medical Physics, Faculty of Medicine, Institute of Biomedical Sciences, Vilnius University, 03101 Vilnius, Lithuania; ingrida.zeleckiene@santa.lt (I.Z.); mindaugas.mataciunas@santa.lt (M.M.)

**Keywords:** pulmonary hypertension, COVID-19, pulmonary fibrosis

## Abstract

*Background and Objectives.* COVID-19 infection has a significant burden on global morbidity and mortality, especially in elderly people and in patients with chronic respiratory and cardiovascular diseases, such as pulmonary hypertension (PH). We aimed to evaluate the impact of COVID-19 infection on patients diagnosed with severe chronic PH. *Materials and Methods.* A single-center prospective cohort study was performed. Patients were enrolled from 1 November 2020 to 31 December 2022. Follow-up was until 31 December 2023. Data were collected on PH diagnosis, clinical presentation, outcomes, brain natriuretic peptide (BNP) levels, pulmonary function test with lung diffusion capacity for carbon monoxide (DLCO), and computed tomography pulmonary angiography (CTPA) analysis. *Results.* During the 26 months of our study, 51 PH patients were diagnosed with COVID-19 infection. The majority, 44 (86.3%) of all COVID-19 infected patients, were treated on an outpatient basis, and 7 (13.7%) required hospitalization. During the follow-up period, 8 (15.7%) patients died: 4 (7.8%) due to complications of COVID-19 infection, and the other 4 (7.8%) died in the later stages of the follow-up period after recovery from acute COVID-19 infection. Therefore, the in-hospital mortality in our study was 43% (*n* = 3). As mentioned above, the overall mortality was 7.8% (*n* = 4). Higher BNP levels in the third month after COVID-19 were associated with higher mortality rates (*p* = 0.028). Lung function, including DLCO, did not significantly worsen with COVID-19. In our study, 24 patients (47.1%) were referred for a follow-up CTPA scan and one of them developed typical fibrotic lung changes after COVID-19. *Conclusions.* The incidence of COVID-19 infection in patients diagnosed with PH was 34%. In our patients with severe chronic PH, the overall mortality rate due to COVID-19 infection was low. Pulmonary fibrosis was a rare complication in our cohort. COVID-19 infection in severe PH may increase the risk of worsening chronic heart failure.

## 1. Introduction

Pulmonary hypertension (PH) is a severe chronic pathophysiological disorder, characterized by pulmonary vascular involvement that can lead to right ventricular failure. PH is highly heterogeneous and can complicate many cardiovascular, respiratory, and systemic diseases [1]. Because PH is associated with worsening symptoms and higher mortality rates, regardless of the underlying condition [2], patients with PH require ongoing care from a variety of healthcare professionals. Pulmonary arterial hypertension (PAH) and chronic thromboembolic pulmonary hypertension (CTEPH) are rare, but are the most severe clinical groups of PH. For this reason, the European Society of Cardiology (ESC) and European Respiratory Society (ERS) guidelines recommend that PAH and CTEPH should be managed by healthcare professionals with specific expertise in these conditions [1]. The complexity of managing PAH/CTEPH patients requires a multidisciplinary approach and highly specialized knowledge, and it is recommended that these patients are treated and monitored in dedicated PH centers [1]. Over the past few decades, significant progress has been made in the recognition and treatment of these diseases. As a result, although PAH and CTEPH are progressive, incurable conditions, in the majority of cases, they are treatable.

In 2020, global health was abruptly destabilized by the sudden emergence and rapid spread of severe acute respiratory coronavirus 2 (SARS-CoV-2), causing the coronavirus disease of 2019 (COVID-19) [3]. By 10 March, >48,000 confirmed cases and ~3000 deaths had been reported worldwide. Finally, the WHO declared COVID-19 as a pandemic on 11 March 2020 [4]. Risk factors for developing severe COVID-19, especially before vaccine availability, were older age and comorbidities, such as chronic cardiovascular and pulmonary disease. Although several early surveys and case reports seemed to indicate that clinical outcomes in PAH patients with COVID-19 were favorable or no worse than in the general population [5,6], later studies with larger sample sizes showed that precapillary PH in COVID-19 patients was associated with high in-hospital mortality, a high hospitalization rate of 60%, and higher mortality and mechanical ventilation rates [7,8,9].

At the beginning of the COVID-19 pandemic, there was a lack of information regarding the impact of COVID-19 infection on individuals with PAH or CTEPH. Therefore, our PH center team decided to follow up even more precisely than usual on those PAH/CTEPH patients who had COVID-19 infection, and to conduct a prospective observational study evaluating the impact of COVID-19 infection on exercise tolerance, lung structural and functional changes, and prognosis.

The main objective of this study was to evaluate the clinical course, treatment, hospitalizations, and outcomes of COVID-19 infection in PAH/CTEPH patients from our Pulmonary Hypertension Referral Center (PHrc) at Vilnius University Hospital Santaros Klinikos (VUHSK). We also aimed to evaluate the incidence of pulmonary fibrosis after COVID-19 infection. Mortality rates and incidence of COVID-19 infection vary between countries.

## 2. Materials and Methods

### 2.1. Study Design

A single-center prospective cohort study was conducted at the Pulmonary Hypertension Referral Centre (PHrc) a member of European Reference Network-LUNG (https://ern-lung.eu/) of Vilnius University Hospital Santaros Klinikos (VUHSK). All adult PH patients diagnosed with COVID-19 infection (positive for SARS-CoV-2 by PCR and SARS-CoV-2 antigen-based ELISA) from 1 November 2020 to 31 December 2022 were enrolled. The follow-up period was until 31 December 2023. This study was approved by the Vilnius Regional Biomedical Research Ethics Committee of Lithuania (protocol code no. 2020/1-1182-669, approval date 28 January 2020). Written informed consent was obtained from all participants.

### 2.2. Data Collection

The following basic demographic and characteristic data were collected: type of PH, comorbidities, type of advanced PH treatment, World Health Organization functional class (WHO-FC), information on vaccination against COVID-19, clinical course of COVID-19 disease, and outcomes. Death was defined in two categories: death due to SARS-CoV-2 infection (COVID-19 infection as the main cause of death) and death in the later stages of follow-up, after recovery from acute COVID-19 infection (pre-existing disease as the main cause of death). The last routine follow-up examinations before COVID-19 infection were collected retrospectively. Prospectively, at 3 and 6 months after COVID-19 infection, patients underwent the following tests: complete blood count, brain natriuretic peptide (BNP), arterial blood gas analysis, pulmonary function test (PFT) with lung diffusion capacity for carbon monoxide (DLCO), and computed tomography pulmonary angiography (CTPA).

### 2.3. Patients

**Figure 1:**
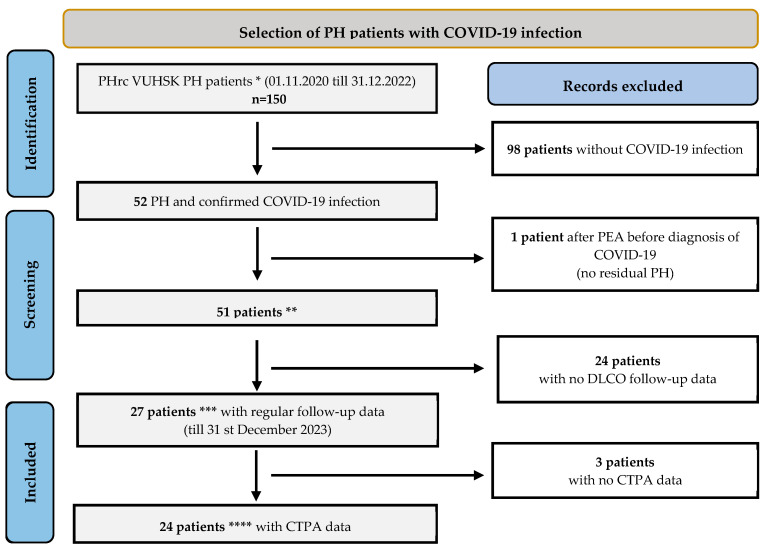
Patient inclusion process. * Patients on close follow-up at the PHrc, ** or demographic and clinical characteristic data analysis, *** for DLCO analysis, **** for CTPA rescan analysis.

We identified 51 patients with PH and COVID-19 infection (Figure 1). All were included in the analysis of demographic and characteristic data. Due to the pandemic, especially during the first few months, regular follow-up visits or scheduled face-to-face consultations were not always available for all patients. We divided the patients into three different subgroups based on the accessibility of the follow-up data: 1: WHO-FC, 6-min walk test (6-MWT) distance and BNP level evaluation subgroup, 2: PFT with DLCO data analysis subgroup (patients who underwent the test in the 12 months before COVID-19 (baseline) and at 3 and 6 months after COVID-19), and 3: the CTPA data analysis subgroup (patients who underwent CTPA in the 12 months before COVID-19 (baseline) and at 3 and 6 months after COVID-19).

### 2.4. CTPA Analysis

CTPA was performed on a 256-slice multidetector CT GE Revolution (Milwaukee, WI, USA), collimation 0.625 mm. Contrast was injected via the antecubital vein at a rate of 4–5 mL/s using Omnipaque 350.

### 2.5. Six-MWT and DLCO Analysis

The 6-MWT was performed according to ATS standards [10]. Pulmonary function testing was performed using the Vmax Encore (Viasys^®^ Healthcare, Yorba Linda, CA, USA). The procedure followed was according to the American Thoracic Society/European Respiratory Society guidelines. DLCO was measured using the single-breath method. The lower limit of normal (LLN) was defined as the 5th percentile according to the Global Pulmonary Function Initiative standardized multi-ethnic reference values for spirometry [11].

### 2.6. Statistical Analysis

The data were analyzed using IBM SPSS Statistics version 27. The Kolmogorov–Smirnov test was used to test the distribution of the data. Categorical variables were presented as absolute numbers and percentages; continuous variables were presented as medians and interquartile ranges [IQRs] or means and standard deviations (±SDs). The Mann–Whitney U test was used for comparisons between groups. Chi-squared or Fisher’s exact test was used to compare categorical variables. Associations were assessed by Spearman’s correlation. The one-way ANOVA test was used to determine whether there were any statistically significant differences between the means of the three DLCO groups. Levene’s test was performed to verify the equality of variances, and the Kruskal–Wallis test was used for the data that were not normally distributed. Statistical significance was defined as a *p*-value of less than 0.05.

## 3. Results

### 3.1. Demographic and Clinical Data

**Figure 2:**
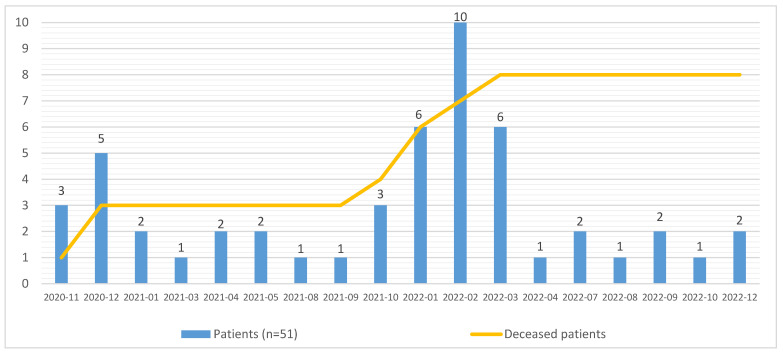
Pulmonary hypertension cases with diagnosed COVID-19 infection.

A total of 150 patients with PH were treated at the VUHSK PHrc during the study inclusion period (Figure 1 and Figure 2). Of these, 34% (*n* = 51) were diagnosed with COVID-19 infection. Two-thirds of them (68.6%) were female (*n* = 35) (Table 1).

The mean age of the infected patients was 58 ± 2.51 years, ranging from 19 to 86 years. More than half of the patients (*n* = 33, 64.7%) had two or more comorbidities, the most common being arterial hypertension (*n* = 32, 62.7%). The majority, 44 (86.3%) of all PH-COVID-19 patients, were treated on an outpatient basis, while 7 (13.7%) required hospitalization. A total of 26 (51%) subjects were vaccinated before COVID-19 infection. The baseline characteristics of our cohort are shown in Table 1.

At baseline, 48 (94%) patients were receiving PH-targeted therapy. Almost half of them were on PH-targeted monotherapy (47.1%, *n* = 24). Dual combination therapy was used in 19 patients (37.3%) and triple combination therapy in 5 patients (9.8%). Balloon pulmonary angioplasty (BPA) was performed in 7 of 17 patients with CTEPH. Three patients (two with idiopathic PAH and one with combined causes) underwent a PH-specific exercise rehabilitation program before the pandemic. Daily supplemental oxygen therapy was administered to seven (13.7%) patients, including six with cyanotic congenital heart disease (five were diagnosed with Eisenmenger’s syndrome) and one patient with multiple PH (PAH/CTEPH/COPD and left heart disease). Three patients underwent COVID-19 infection before the precise PH diagnosis was confirmed; these individuals were later diagnosed with PAH associated with CTD, CTEPH, and combined PH.

### 3.2. WHO-FC, 6-MWT Distance, and BNP Level Evaluation Subgroup

**Figure 3:**
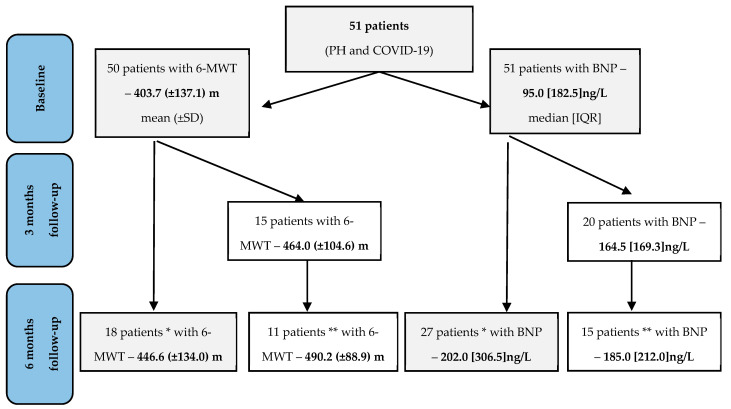
Six-MWT distance and BNP level at baseline, 3 and 6 months at the follow-up. * All patients who had 6-MWT or BNP at 6 months, ** patients who had 6-MWT or BNP at 3 and 6 months.

At baseline (last visit to PHrc before diagnosis of COVID-19 infection), the majority of cases (*n* = 34, 66.7%) were in World Health Organization functional class (WHO-FC) III (Table 1). Deterioration of WHO-FC after 6–12 months of follow-up was observed in 4 of 51 (7.8%) patients (II– > II–III; II–III– > III; II–III– > III–IV and III– > II–IV). Baseline 6-MWT distance results were available for 50 patients, and the mean 6-MWT distance for these patients was 403.7 (±137.1) m (Figure 3).

In our study, only 15 patients had 6-MWT results at 3 months and 18 patients at 6 months. No significant difference was found between the 6-MWT distances at baseline and 3 and 6 months after COVID-19. A decrease in the 6-MWT distance was observed in 7 of 15 (46.7%) and 9 of 18 (50.0%) patients at 3 and 6 months. The mean baseline 6-MWT distance in patients who died (*n* = 8) was shorter (380.0 (±135.7) m) than in those who survived (*n* = 42) (408.2 (±138.6) m).

The median BNP level available for 51 patients was 95.0 [182.5] ng/L (Figure 3). Two-thirds of patients (66.7%—34/51 pts) had an elevated BNP level (>50 ng/L). BNP levels in deceased patients (*n* = 8) were higher at baseline (203.5 [429] ng/L) than in survivors (*n* = 43) (94.0 [153.0] ng/L). There was a weak but significant negative correlation between 6-MWT distance and BNP level at baseline (r = −0.456, *p* = 0.029). An increase in BNP was observed in 14 out of 20 (70.0%) and 16 out of 27 (59.3%) patients at 3 and 6 months, respectively. These patients with worsening BNP levels at 3 months were more likely to have PAH associated with congenital heart disease or PAH/CTEPH combined with left heart disease (28.6% and 28.6%, respectively), were in WHO-FC III (71.4%), and most (92.9%) had an elevated BNP level (>50 ng/L) at baseline. Higher BNP levels at 3 and 6 months after COVID-19 were associated with higher mortality during the later follow-up period (*p* = 0.028 and *p* = 0.047, respectively).

### 3.3. Diffusing Capacity of the Lungs for Carbon Monoxide Evaluation Subgroup

Twenty-seven patients were included in the gas diffusion assessment (Table 2). In 7 of them, the gas diffusion test was performed three times—at baseline, after 3 and after 6 months (group I); 13 subjects underwent DLCO only at baseline and after 3 months (group II), and another 7 at baseline and after 6 months (group III).

At baseline, approximately half of all patients—14 (51.9%)—had moderately impaired DLCO, and 1 patient (3.7%) had severely impaired DLCO (26%).

### 3.4. Computed Tomography Pulmonary Angiography (CTPA) Subgroup

**Figure 4:**
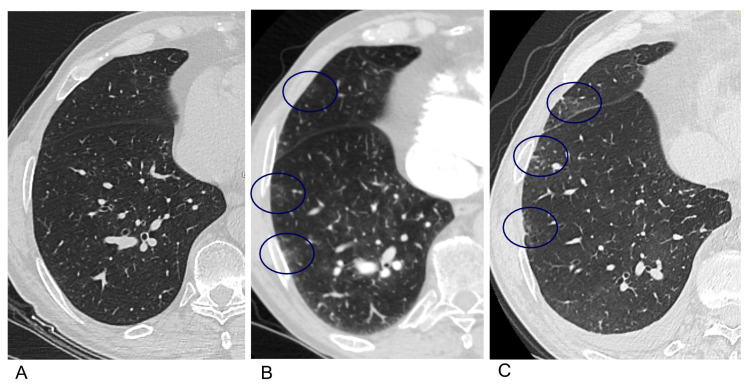
Evolution of chest computed tomography pulmonary angiography (CTPA) features before and after COVID-19 pneumonia in a patient with pulmonary hypertension. (**A**) Chest CTPA image (lung window) before COVID-19 pneumonia, no changes in the lung parenchyma. (**B**) Two-month follow-up CTPA after COVID-19 pneumonia: subtle ground-glass opacities in the periphery of the parenchyma (circles). (**C**) Follow-up CTPA after 2 years: typical fibrotic lung changes after COVID-19 (subpleural reticulations with subtle ground glass in the basal lung parts (circles)).

In our study population, 24 (47.1%) patients were referred for follow-up CTPA scans and only one of them showed typical fibrotic lung changes after COVID-19 (subpleural reticulations with subtle ground-glass nodules in the basal parts of both lungs (Figure 4)). The remaining patients had previously known lung parenchymal changes not associated with COVID-19 infection (*n* = 8, 33.3%) or no characteristic lung parenchymal changes at all (*n* = 15, 62.5%). Lung parenchymal changes that did not show progression at follow-up (CTPA images 6 months after COVID-19 infection) were as follows. Significant pulmonary fibrosis was detected in one patient with PAH associated with connective tissue disease (CTD). Two patients (8.3%) had CTEPH and we observed typical ground-glass nodules in both lungs on their CTPA scans, representing regional impairment of pulmonary perfusion. Five patients (20.8%) had typical ground-glass nodules on CTPA images. No new cases of CTEPH were identified after COVID-19.

### 3.5. Outcomes

Of the total cohort, 44 (86.3%) patients were treated on an outpatient basis, while 7 (13.7%) patients had a severe course of COVID-19 infection requiring hospitalization. During the follow-up period, eight patients (15.7%) died: four patients (7.8%) died as a result of complications of COVID-19 infection (three in hospital and one at home), and the other half of the patients (*n* = 4, 7.8%) died of causes unrelated to COVID-19 infection in the later follow-up period after the COVID-19 infection had resolved (one from sepsis, two from worsening chronic heart failure and one from sudden cardiac death) (Table 3). The mean age of the patients who died from COVID-19 infection was 68.0 years (±16.4), and only one had been vaccinated.

Therefore, the in-hospital mortality in our study was 43% (*n* = 3). As mentioned above, the overall mortality was 7.8% (*n* = 4) (Table 1).

Most of the patients who died had multiple comorbidities. Arterial hypertension and dyslipidemia were recurrent comorbidities in three out of four patients in the group where COVID-19 infection was the main cause of death. Two patients in this group had hematological disorders (chronic myeloid leukemia and chronic B-cell lymphoma). Three patients were in WHO-FC III and one in WHO-FC III-IV.

A total of eight patients died, four on PH monotherapy, three on double therapy, and one on triple therapy (Table 3).

## 4. Discussion

The sudden and unexpected global spread of the COVID-19 virus in early 2020 has hurt the health of both previously healthy people and those with chronic diseases, as well as impacting on the provision of health services. Fortunately, the variability of COVID-19 infection within European countries has been very different. The peak of COVID-19 infection occurred later in Lithuania [12]. About half of our patients (51%) were vaccinated at that time. Less than a third (31.4%) contracted COVID-19 infection before the vaccine was available in our country and refused vaccination, claiming that their history of COVID-19 infection protected them.

There is a lack of literature on how many PH patients were vaccinated at the same time in other countries, but comparing data from neighboring Poland, they had higher vaccination rates: 77% of PAH/CTEPH patients were vaccinated by the end of September 2021, and only 15% were convinced that their history of COVID-19 sufficiently protected them against reinfection [13].

COVID-19 infection is most likely to affect the lungs. In addition, viral infection may cause alveolar septal fibroproliferation and pulmonary consolidation [14]. Risk factors for developing severe COVID-19, especially before vaccine availability, are older age and comorbidities such as chronic cardiovascular and pulmonary disease [15].

Patients with chronic PH have been among the hardest hit by the COVID-19 pandemic. Not only has the COVID-19 pandemic disrupted the provision of essential regular healthcare services to these heavily ill PAH/CTEPH patients [16,17,18], but the mortality rates associated with COVID-19 infection in this specific population are high, although variable in different sources, ranging from overall mortality of 9.5% to 28% [8,9,17,19,20,21,22,23] and in-hospital mortality of 21.4% to 45.5% [7,8,9,19,22,23]. The results of our center’s PH cohort show a relatively low all-cause mortality rate (7.8%) and a high in-hospital mortality rate (43%).

While the case-fatality rates due to COVID-19 rates appear to increase in PAH/CTEPH patients compared to the general population, conflicting data exist across different countries. For instance, a survey by Lee et al. calculated a 12% COVID-19 case-fatality rate in PAH/CTEPH patients in the United States [20], while other international surveys from 28 countries revealed the rate to be 19% [17]. In Greece, Farmakis et al. reported a 22% COVID-19 mortality in their PAH/CTEPH population [22]. The French cohort showed an overall case-fatality of 24.6%, where male gender, older age, comorbidities, and more severe PH were associated with increased mortality risk [9]. Similar to the Greek and French data, the PH referral center in Brazil reported a 23% case-fatality rate associated with COVID-19 infection in PH patients [21]. Conversely, a publication from neighboring Poland indicated an 8% case-fatality rate due to COVID-19 in PAH/CTEPH patients during the 2-year pandemic [23] aligning more closely with our results. In addition, a single-center prospective study from Spain reported a 9.5% overall mortality in their PAH/CTEPH patients [19].

The higher BNP at 3 and 6 months after COVID-19 was associated with higher late mortality. Certainly, elevated BNP levels were associated with PH and progression to heart failure. However, it remains unclear whether the deterioration was due to the COVID-19 infection itself or to the underlying PH, which is characterized by a progressive course despite treatment, or even to other factors, such as reduced access to health services. Future multicenter observational studies would be beneficial.

The hospitalization rate for COVID-19 in our cohort was 13%—also comparatively lower than rates reported in the United States (30%), Greece (44%), France (60%), Brazil (31%), Poland (31%), or Spain (44%) [9,19,20,21,22,23]. It is plausible that a larger proportion of asymptomatic or mildly symptomatic cases were identified in our cohort, given the extensive COVID-19 testing programs implemented in Lithuania during the period of our study [24]. Furthermore, despite the challenges faced by many people in accessing timely medical care due to the burden of COVID-19 on public health systems, patients in our PH referral center received continuous expert care during this period. At least remote consultations by telephone with extended individualized care recommendations were accessible to our PH patients, even during their COVID-19 infection. In addition, PH patients and their caregivers tend to be cautious and well-informed about their health due to the long period of the chronic disease and the higher risk of infection during their lifetime. This probably contributed to higher adherence to recommendations for isolation, social distancing, and general prevention of airborne infections during the pandemic. Finally, there have been hypotheses suggesting a potential beneficial effect of PAH-specific therapies for COVID-19 disease, due to their protective effect on the endothelium [25]. Some even theorize that the disease itself may confer protective qualities, as PAH patients have reduced expression of angiotensin-converting enzyme 2—a critical entry receptor for the COVID-19 virus [26]. However, these speculations remain largely theoretical and require further rigorous studies for validation.

Assessment of pulmonary gas diffusion is one of the most important indicators of the impact of COVID-19 infection on respiratory function. Previous studies in the general population have shown a significant improvement in lung function at follow-up, particularly in severe forms of the disease [27,28,29]. According to a study published by Kristyn L. Lewis [30] analyzing pre- and post-infection lung function parameters, lung function tests in individuals who recover from COVID-19 without intubation or positive pressure ventilation are likely to return to pre-infection parameters. According to one study, chest CT scores for pulmonary fibrosis decreased dramatically in the majority of COVID-19 patients one, two, and three months after discharge, suggesting that pulmonary fibrosis is likely to decrease over time after COVID-19 [31]. This is confirmed by our results: only one CTEPH patient had significant progression of lung fibrosis.

However, data on the impact of COVID-19 infection on lung function parameters, especially DLCO, in patients with pre-existing PH are severely lacking.

Our longitudinal analysis of lung function impairment 6 months after COVID-19 infection in PH patients showed no significant difference in DLCO. This suggests that COVID-19 infection did not significantly affect gas diffusion parameters in the PH patients we studied, but larger cohort studies are needed.

According to recent studies, pulmonary fibrosis after COVID-19 infection is not common, accounting for 7% of cases [32,33]. In our study, five patients (9.8%) had typical ground-glass nodules on CT images, described in the literature as cholesterol granulomas after repeated bleeding [34].

Our evaluation of the results should be interpreted with caution due to the small total number of patients. Not all statistica parameters have been calculated due to the small sample size. In addition, the COVID-19 pandemic posed a challenge to our data collection efforts, as regular follow-up or scheduled “face-to-face” consultations were not available for all patients, especially in the early months, resulting in missing data. In addition, we cannot exclude the possibility that some patients had undiagnosed COVID-19 infection due to a lack of symptoms. It is also important to note that our study was conducted in a single PH referral center, which may limit the generalizability of the findings to other populations or healthcare settings. In addition, the limited follow-up period after COVID-19 may not have captured longer-term effects or changes in outcomes.

## 5. Conclusions

The incidence of COVID-19 infection in patients diagnosed with PH was 34% at the PH referral center of Vilnius University Hospital. Fortunately, the overall mortality rate due to COVID-19 infection was low in our patients with severe chronic pulmonary hypertension. Pulmonary fibrosis was a rare complication in our cohort. COVID-19 infection did not significantly worsen lung function in our patients. COVID-19 infection in severe PH may increase the risk of worsening chronic heart failure.

## Figures and Tables

**Table 1:** Basic characteristics of patients with pulmonary hypertension and COVID-19. Data comparison between the groups depending on the survival.

Variable	Dead Due to COVID-19 * (*n* = 4)	Survivors (*n* = 43)	*p*-Value	Total (*n* = 51)
**Age, years ± SD**	68.0 ± 16.4	57.0 ±17.9	0.511	58.0 ± 2.5
**Weight, kg ± SD**	97.8 ± 35.7	74.2 ±19.2	0.511	76.4 ± 21.1
**Females, *n* (%)**	2 (50.0)	30 (69.8)	0.583	35 (68.6)
**Baseline WHO functional class, *n* (%)**			0.755	
-I–II	0	2 (4.7)		2 (3.9)
-II	0	6 (14.0)		6 (11.8)
-II–III	1 (25.0)	6 (14.0)		9 (17.7)
-III	3 (75.0)	29 (67.4)		34 (66.7)
**Type of PH (*n*, %)**			0.587	
-Idiopathic PAH	1 (25.0)	7 (16.3)		9 (17.7)
PAH				
-associated with CTD	1 (25.0)	5 (11.6)		6 (11.8)
-associated with CHD	0	11 (25.6)		12 (23.5)
CTEPH	1 (25.0)	15 (34.9)		17 (33.3)
Other PH ^&^	1 (25.0)	5 (11.6)		7 (13.7)
**Comorbidities, *n* (%)**				
-Arterial hypertension	3 (75.0)	27 (62.8)	0.541	32 (62.8)
-Coronary artery disease	1 (25.0)	9 (20.9)	0.670	12 (23.5)
-Dyslipidemia	3 (75.0)	17 (39.5)	0.298	22 (43.1)
-Atrial fibrillation/flutter	1 (25.0)	8 (18.6)	0.586	10 (19.6)
-COPD	0	4 (9.3)	0.692	4 (7.8)
-Anemia	2 (50.0)	17 (39.5)	0.536	19 (37.3)
-2 comorbidities	0	15 (34.9)	0.446	15 (29.4)
-3 comorbidities	1 (25.0)	6 (14.0)		8 (15.7)
-4 comorbidities	2 (75.0)	6 (14.0)		9 (17.7)
-5 comorbidities	0	1 (2.3)		1 (2.0)
**Vaccination, *n* (%)**	1 (25.0)	24 (55.8)	0.328	26 (51.0)
**Advanced PH treatment, *n* (%)**			0.703	
-No	0	3 (7.0) ^#^		3 (5.9) ^#^
-Monotherapy	3 (75.0)	20 (46.5)		24 (47.1)
-Dual combination therapy	1 (25.0)	16 (37.2)		19 (37.3)
-Triple combination therapy	0	4 (9.3)		5 (9.8)
**The hospitalization due to COVID-19, *n* (%)**	3 (75)	3 (7)	0.008	7 (13.7)
**Baseline 6 MWT, m ± SD**	337.5 ± 113.2	408.2 ± 138.6	0.511	403.7 ± 137.1
**Baseline BNP, ng/L [IQR]**	168.5 [94.8]	94 [180]	0.623	95.0 [182.5]

Categorical data are shown as *n* (%). Continuous data are depicted as median and interquartile ranges [IQRs] or means and standard deviations (±SDs). WHO—World Health Organization, PAH—pulmonary arterial hypertension, PH—pulmonary hypertension, CTEPH—chronic thromboembolic pulmonary hypertension, CTD—connective tissue disease, CHD—congenital heart disease, COPD—chronic obstructive pulmonary disease, SD—standard deviation. * Deaths not related to COVID-19 were excluded. ^&^ PAH/CTEPH in combination with COPD or left heart disease. ^#^ These patients underwent COVID-19 infection before precise PH diagnosis was confirmed; later, PAH associated with CTD, CTEPH, and other PH were diagnosed for these patients.

**Table 2:** Gas diffusion at baseline, 3 and 6 months at the follow-up.

Group	DLCO (%) Baseline Mean (± SD)	DLCO (%) 3 Months Mean (± SD)	DLCO (%) 6 Months Mean (± SD)	*p*-Value
I (*n* = 7)	55.0 (±5.9)	59.7 (±10.2)	64.4 (±7.6)	0.12 *^#^
II (*n* = 13)	63.8 (±14.4)	62.1 (±12.1)	-	0.73 *^#^
III (*n* = 7)	59.7 (±19.7)	-	56.4 (±19.1)	0.75 *^#^

Data are expressed as mean ± standard deviation (SD). DLCO—gas diffusion of carbon monoxide. * One-way ANOVA showed no significant difference in DLCO parameters between groups. ^#^ Kruskal–Wallis test showed no statistically significant difference between groups.

**Table 3:** The baseline characteristics of deceased patients.

Age (Years)	Sex	Main Diagnosis	Comorbidities	Functional Class	6-MWT	BNP ng/L	PH Treatment	Cause of Death	Vaccinated against COVID-19
	**COVID-19 infection as the main cause of death**								
63	Female	CTEPH and left heart disease	AH, dyslipidemia, obesity, chronic AF.	II–III	240	272	Sildenafil	COVID-19	No
47	Male	CTEPH	AH, dyslipidemia, obesity, sleep apnoea, chronic myeloid leukemia, anemia	III	450	65	Sildenafil *	COVID-19	Yes
80	Male	IPAH	AH, dyslipidemia, CAD, chronic B-cell lymphoma, sick sinus syndrome	III	420	34	Sildenafil Macitentan	COVID-19	No
82	Female	PAH-CTD	Anaemia	III	240	543	Sildenafil	COVID-19	No
	**Pre-existing disease as the main cause of death** (deaths in the later stages of the follow-up period, post-recovery from acute COVID-19 infection)								
39	Female	IPAH	Multiple myeloma	II–III	605	135	Sildenafil Bosenatn Selexipag	Sepsis and MODS	No
78	Male	CTEPH	AH, dyslipidemia, CAD, chronic AF, chronic kidney disease, gout	III	375	871	Sildenafil Ambrisentan	CHF deterioration	No
76	Female	CTEPH and left heart disease	AH, dyslipidemia, CAD, chronic AF, chronic kidney disease, gout	III	230	293	Sildenafil	CHF deterioration	No
43	Female	PAH-CHD (Eisenmenger’s syndrome: TGA and single ventricle)	Ventricular extrasystoles	II–III	480	47	Sildenafil Ambrisentan	Sudden cardiac death	Yes

* Due to a hematological disorder, this patient was treated with imatinib. AH—arterial hypertension, AF—atrial fibrillation, CAD—coronary artery disease, MODS—multiple organ dysfunction syndrome, TGA—transposition of the great arteries, CTEPH—chronic thromboembolic pulmonary hypertension, IPAH—idiopathic pulmonary arterial hypertension, PAH-CTD—pulmonary arterial hypertension—connective tissue disease, CHF—chronic heart failure.

## Data Availability

The data that support the findings of this study are available on request from the corresponding author (V.R.).
